# Correction: Interpersonal responses to facial expressions of disgust, anger, and happiness in individuals with varying levels of social anxiety

**DOI:** 10.1371/journal.pone.0283038

**Published:** 2023-03-09

**Authors:** The PLOS ONE Staff

## Notice of Republication

This article was republished on January 17, 2023, to correct for errors in the Data Availability statement. The publisher apologizes for the errors. Please download this article again to view the correct version. The originally published, uncorrected article and the republished, corrected article are provided here for reference.

## Supporting information

S1 File Originally published, uncorrected article. (PDF)

S2 File Republished, corrected article. (PDF)
