# Supplementary material for: Interpersonal responses to facial expressions of disgust, anger, and happiness in individuals with varying levels of social anxiety
Source: PLoS One. 2022 Apr 7;17(4):e0263990. doi: 10.1371/journal.pone.0263990 (PMC8989355; doi:10.1371/journal.pone.0263990)
Supplement: Attachment — Submitted filename: Response to Reviewers.docx [file pone.0263990.s001.docx]

**Response to Reviewers**

Reviewer 1

*Comment 1:* In the Results section, the test significance (i.e., p) should be reported also in the text, together with descriptive data, such as mean and standard deviation (e.g., in the first sentences of the paragraphs Communion and Agency of the Results section).

*Response 1:* All means and standard errors provided in Table 2 (formerly Table 1, see Response 4 below) are now also reported in the text, see pages 17-21. The significance of *F* tests provided in Tables 3 and 4 (formerly Tables 2 and 3) for the main effects of Expression and the Expression by Intensity, Expression by BFNE, and Expression by SIAS interaction terms are now also reported in the text.

*Comment 2:* In the Measures section, more information should be reported, specifically: the total score, the total number of items, and the direction of the scale (i.e., what a greater score means) of the Brief Fear of Negative Evaluation (BFNE) scale; the total number of items of the Social Interaction Anxiety Scale (SIAS); the total score, the direction of the scale, and the exact number of items for each subscale (it is not clear if the total items are equally divided between the two subscales) of the Revised Disgust Propensity and Sensitivity Scale (DPSS-R).

*Response 2:* For the BFNE and SIAS, we now also report the total number of items. For the BFNE, we now also report the minimum and maximum total score, and the direction of the scale. See page 11. The 16 items of the DPSS-R are equally divided over the two subscales; this has now been clarified on page 11. Also, the minimum and maximum subscale scores and the direction of both subscales are now reported in this section, see page 12.

*Comment 3:* Also, in the Measures section, internal consistency and correlational results should be moved in the Results section.

*Response 3:* Data on the internal consistency of the BFNE and SIAS and how they were correlated has now been moved to the Results section, see page 18.

*Comment 4:* The Revised Disgust Propensity and Sensitivity Scale (DPSS-R) seems not to be included in any of the analyses. However, the DPSS-R mean and standard deviation could be relevant to be reported. The authors could consider adding an additional table showing demographic characteristics of the sample as well as DPSS-R scores.

*Response 4:* We now include a Table 1 that provides descriptive information about the sample, including DPSS-R scores. See pages 14-15. Due to the inclusion of this new table, subsequent tables are now numbered Tables 2-4 (formerly Tables 1-3).

Journal requirements

Comment 1: Please ensure that your manuscript meets PLOS ONE's style requirements, including those for file naming.

Response 1: We now use the required file names.

Comment 2: Please note that according to our submission guidelines, outmoded terms and potentially stigmatizing labels should be changed to more current, acceptable terminology. To this effect, “Caucasian” should be changed to “white” or “of [Western] European descent” (as appropriate)

Response 2: Throughout the manuscript we changed “Caucasian” to “of Western European descent”, see pages 9-10 specifically.

Comment 3: Any changes to the reference list should be mentioned in the rebuttal letter that accompanies your revised manuscript.

Response 3: There have been no changes in the reference list.
